# Evidence for a role of adaptive immune response in the disease pathogenesis of the MPTP mouse model of Parkinson's disease

**DOI:** 10.1002/glia.22935

**Published:** 2015-10-29

**Authors:** Heather L. Martin, Matteo Santoro, Sarah Mustafa, Gernot Riedel, John V. Forrester, Peter Teismann

**Affiliations:** ^1^ Institute of Medical Sciences, Foresterhill, University of AberdeenAberdeenAB25 2ZDUnited Kingdom; ^2^ Ocular Immunology Program, Centre for Ophthalmology and Visual Sciencethe University of Western AustraliaWestern Australia6009Australia; ^3^ Centre for Experimental ImmunologyLions Eye InstituteNedlandsWestern Australia6009Australia

**Keywords:** microglia, Parkinson's disease, MHCII, MPTP, neuroinflammation

## Abstract

Parkinson's disease (PD) is the second most common neurodegenerative disease and results from the loss of dopaminergic neurons of the nigrostriatal pathway. The pathogenesis of PD is poorly understood, but inflammatory processes have been implicated. Indeed increases in the number of major histocompatibility complex II (MHC II) reactive cells have long been recognised in the brains of PD patients at post‐mortem. However whether cells expressing MHC II play an active role in PD pathogenesis has not been delineated. This was addressed utilising a transgenic mouse null for MHC II and the parkinsonian toxin 1‐methyl‐4‐phenyl‐1,2,3,6‐tetrahydropyridine (MPTP). In wild‐type mice MHC II levels in the ventral midbrain were upregulated 1–2 days after MPTP treatment and MHC II was localized in both astrocytes and microglia. MHC II null mice showed significant reductions in MPTP‐induced dopaminergic neuron loss and a significantly reduced invasion of astrocytes and microglia in MHC II null mice receiving MPTP compared with controls. In addition, MHC II null mice failed to show increases in interferon‐γ or tumour necrosis factor‐α in the brain after MPTP treatment, as was found in wild‐type mice. However, interleukin‐1β was significantly increased in both wild‐type and MHC II null mice. These data indicate that in addition to microglial cell/myeloid cell activation MHC Class II‐mediated T cell activation is required for the full expression of pathology in this model of PD. GLIA 2016;64:386–395

## Main Points

- MHCII upregulation is necessary for dopaminergic cell death in a model of Parkinson's disease. The study supports the presence of an adaptive immune response in the pathogenesis of Parkinson's disease.

**Abbreviations**
IFNγ: Interferon‐γ
IL‐1β: Interleukin‐1β
MPTP: 1‐methyl‐4‐phenyl‐1,2,3,6‐tetrahydropyridine
TNFα: Tumour necrosis factor‐α

## Introduction

Parkinson's disease (PD) is the second most common neurodegenerative disease (Dauer and Przedborski, 2004), affecting 120,000 people in the UK alone, with 10,000 new cases per annum. Its primary neuropathological feature is the loss of dopaminergic nigrostriatal neurons (Dauer and Przedborski, 2004). The pathogenesis of this debilitating disease is poorly understood (Dauer and Przedborski, 2004), but inflammatory processes have been implicated in the degeneration of the dopaminergic neurons. This is supported by the activated glial cells and the upregulation of pro‐inflammatory cytokines seen in both models of PD and PD patients (Czlonkowska et al., 1996; Hebert et al., 2003; McGeer et al., 1988; Mogi et al., 1994a,b).

Major histocompatibility complex class II (MHC II) molecules present endocytosed antigens to CD4^+^ T‐helper cells (Cresswell, 1994). Under normal conditions the central nervous system expresses low levels of MHC II (Shrikant and Benveniste, 1996); however increases in MHC II levels have been documented in a number of pathological states including multiple sclerosis (Hofman et al., 1986) and Alzheimer's disease (Parachikova et al., 2007). Increases in MHC II‐positive cells have long been recognised in human post‐mortem tissue from PD patients (Imamura et al., 2003; McGeer et al., 1988). Also an increase in the number of MHC II‐positive microglia is seen in mice treated with 1‐methyl‐4‐phenyl‐1,2,3,6‐tetrahydropyridine (MPTP), a drug which induces a PD‐like disease in mice (Kurkowska‐Jastrzebska et al., 1999a, 1999b). A role for MHC II in PD pathogenesis is further supported by the presence of an infiltrate of CD4^+^ T‐cells in PD patients (Brochard et al., 2009), as recruitment of CD4^+^ T‐cells requires MHC II signalling (Cresswell, 1994). Indeed mice null for CD4^+^ T‐cells are protected from MPTP toxicity (Brochard et al., 2009). Despite this body of evidence suggesting that MHC II plays a role in the degeneration of dopaminergic neurons direct evidence for the role of MHC II in PD pathogenesis has not been demonstrated. This study aims to determine whether MHC II is required for PD pathogenesis by utilising a transgenic mouse null for MHC II and the parkinsonian toxin MPTP.

## Materials and Methods

### Animals and Treatments

All procedures were in accordance with the Animals (Scientific Procedures) Act 1986 and MPTP handling and safety measures were consistent with (Jackson‐Lewis and Przedborski, 2007). Twelve week‐old male C57BL6 mice (Charles River Laboratories, UK) or MHC II null mice, previously described by (Lau et al., 2008) received intraperitonal injections of MPTP‐HCl (30mg/kg free base; Sigma Aldrich, Poole, UK) dissolved in saline, one injection for five consecutive days, and were killed at selected times ranging from 0 to 21 days after the last injection. Control mice received saline only.

### MHC II, Tyrosine Hydroxylase (TH), Glial Fibrillary Acid Protein (GFAP) and Ionized Calcium‐Binding Adaptor Molecule‐1 (Iba1) Immunohistochemistry

Immunofluorscent staining was performed as described in (Teismann et al., 2003). Primary antibodies were rat anti‐MHC II (1:200; eBioscience, Hatfield, UK), mouse anti‐TH (1:500; Chemicon, Temecula, CA), mouse anti‐human GFAP (1:100; DAKO, Cambridgeshire, UK) and rabbit anti‐Iba‐1 (1:1000; Wako Chemicals, Neuss, Germany). Immunostaining was visualized with Alexa Fluor 488 anti‐rabbit (1:300; Molecular Probes, Eugene, OR), Alexa Fluor 488 anti‐rat (1:300; Molecular Probes) cy‐3 anti‐mouse (1:200; Jackson Immuno Research, West Grove, PA), cy‐3 anti‐rabbit (1:200; Jackson Immuno Research) and confocal microscopy (LSM 510, Carl Zeiss, Hertfordshire, UK).

Immunostaining for stereological counting of TH‐ and Nissl‐stained neurons in the substantia nigra pars compacta (SNpc) was carried out on midbrain sections as described in (Teismann et al., 2003) using a polyclonal rabbit anti‐TH (1:1000; Chemicon) and visualized with 3,3′‐diaminobenzidine (SigmaAldrich). The sections were counted using regular light microscopy (AxioImager M1, Carl Zeiss) and the optical fractionator method (West, 1993) (Stereo Investigator version 7, MBF Bioscience, Magdeburg, Germany). Stereological counting of microglia and astrocytes in the SNpc was carried out on midbrain sections as described for TH‐stained neurons using a rabbit anti‐Iba1 (1:1,000; Wako Chemicals) for microglia and a rabbit anti‐ GFAP antibody (1:500; DAKO) for astrocytes. A mouse anti‐TH was used to permit the SNpc to be identified (1:1,000; Chemicon). Staining was visualized using cy‐3 anti‐rabbit (1:300; Jackson Immuno Research) and cy‐2 anti‐mouse (1:300; Jackson Immuno Research) antibodies. The sections were counted using fluorescence microscopy (AxioImager M1) and the optical fractionator method (Stereo Investigator version 7).

### RNA Extraction and Quantitative RT‐PCR

Total RNA was extracted from selected brain regions using the TRIzol (Invitrogen) homogenization method as in the manufacturer's instructions. Samples were then subjected to a DNase digestion, DNase I Amp Grade kit (Invitrogen), as per manufacturer's instructions. First strand cDNA synthesis was carried out using the Superscript II kit (Invitrogen). The primer sequences used in this study were MHC II β‐chain 5′‐ ACACGGTGTGCAGACACAA‐3′ (forward), 5′‐TCAGGCTGGGATGCTCC‐3′ (reverse), β‐actin as 5′‐TGTGATGGTGGGAATGGGTCAG‐3′ (forward) and 5′‐TTTGATGTCACGCACGATTTCC‐3′ (reverse). Quantitative PCR amplification was undertaken using the Lightcycler 480 and the perfecta SYBR Green Fastmix kit (Quanta Biosciences, Gaithersburg, MD) as per the manufacturer's instructions. The identity of fragments amplified with these primers was confirmed by DNA sequencing performed by The Sequencing Service (College of Life Sciences, University of Dundee, Scotland, www.dnaseq.co.uk) using Applied Biosystems Big‐Dye Ver 3.1 chemistry on an Applied Biosystems model 3730 automated capillary DNA sequencer.

### Western Blot Analysis

Total proteins from mouse ventral midbrain, striatum and cerebellum samples were isolated in NP‐40 buffer (20 mM Tris–HCl pH 8; 137 mM NaCl; 10% glycerol; 1% NP‐40; 2 mM EDTA and protease inhibitors (cOmplete Mini EDTA‐free cocktail, Roche)) 1:20 (wt/vol). Protein concentration was determined using a bicinchoninic acid kit (Pierce, Rockford, IL). After boiling in Laemmli's buffer, 20 µg of protein was separated by electrophoresis on a 12% sodium dodecyl sulphate–polyacrylamide gel, transferred to nitrocellulose membrane, and blocked with 2% BSA, 5% or 2% non‐fat dried milk in PBS containing 0.05% Tween‐20 (vol/vol). Overnight incubation with primary antibody at 4°C followed. Primary antibodies were rat anti‐MHC II (1:750; eBioscience), mouse anti‐interferon‐γ (1:500; ThermoScientific, Cambridge, UK), rabbit anti‐tumour necrosis factor‐α (1:200; Abcam, Cambridge, UK), rabbit anti‐interleukin‐1β (1:500; Abcam), and mouse anti‐β‐actin (1:25,000; SigmaAldrich). Blots were then washed a second time in PBS‐Tween (0.05%) and incubated with an appropriate secondary antibody (Jackson Immuno Research). Blots were washed in PBS‐Tween (0.05%) and developed using the Supersignal West Dura kit (Pierce) as per manufacturer's instructions. Bands were visualized with an AlphaInnotech digital imaging system (San Leandro, CA) and quantified with AlphaEase FC 5.02 software.

### HPLC Analysis of Striatal Dopamine and 3,4‐Dihydroxyphenylacetic Acid (DOPAC) Levels

High‐performance liquid chromatography (HPLC) with electrochemical detection was used to measure striatal levels of dopamine, and DOPAC using a method that has been described (Sathe et al., 2012). Briefly, mice were killed, 21 days after the last MPTP injection, and the striata were dissected out and snap frozen on solid carbon dioxide. Striata were then homogenised in 0.1 M perchloric acid (1:30 wt/vol), sonicated and centrifuged at 18,600 x g at 4°C for 20 mins. Following centrifugation 20 μl of sample was injected onto a C18 column (Dionex, Germering, Germany) The mobile phase consisted of 90% 50 mM sodium acetate, 35 mM citric acid, 105 mg/L octane sulfonic acid, 48 mg/L sodium EDTA solution, and 10% methanol at pH 4.3. Flow rate was 1 ml/min. Peaks were detected by an ESA Coulochem II electrochemical detector (ESA, Dionex), and the detector potential was set at 700 mV. Data were collected and processed using the Chromeleon computer system (Dionex).

### Statistical Analysis

Data were analyzed in SigmaPlot 11 for Windows (Systat Software, London, UK). All values are expressed as the mean ± SEM. Normal distribution of the data was tested and the homogeneity of variance confirmed with Levene Test. ANOVA was used to analyse differences among means with time, treatment, or genotype as the independent factor, when the data was normally distributed. When ANOVA showed significant differences Dunnett post‐hoc testing was used in time‐course experiments to compare to saline‐treated mice, in other experiments student Newman–Keuls post hoc testing was used to make pairwise comparisons between means. Data not normally distributed were analyzed with the Kruskal‐Wallis test followed by Mann Whitney U‐tests. The null hypothesis was rejected at the 0.05 level.

## Results

### Effect of MPTP Treatment on MHC II Expression

**Figure 1:**
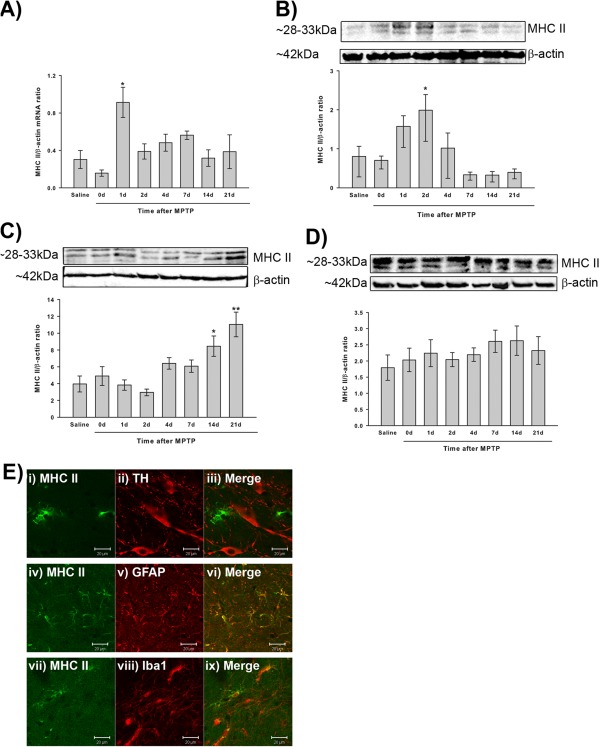
Alterations in MHC II expression and MHC II immunolocalisation following MPTP treatment. MHC II mRNA levels in the ventral midbrain are increased one day after MPTP compared to saline‐treated mice (**A**), with a corresponding increase in MHC II protein two days after MPTP (**B**). In the striatum MHC II protein levels are increased at 14 and 21 days after MPTP treatment (**C**). MHC II protein levels are unchanged in the cerebellum after MPTP treatment (**D**). Data are mean ± SEM, *n* = 3–6 mice per timepoint. *P < 0.05, **P < 0.01 compared to saline (ANOVA with Dunnett's post hoc test) (d—days after MPTP (5 × 30mg/kg) administration). (**E**) Double immunofluorescence of the SNpc confirms that two days after MPTP treatment MHC II (green) is not expressed in TH‐positive neurons (i‐iii; red), but is present in GFAP‐positive astrocytes (iv‐vi; red) and a subset of Iba1‐positive microglia (arrowed) (vii‐ix; red). (TH—tyrosine hydroxylase; GFAP—glial fibrillary acidic protein; Iba1—Ionized calcium‐binding adaptor molecule 1). Scale bars are 20µm. [Color figure can be viewed in the online issue, which is available at wileyonlinelibrary.com.]

MHC II is upregulated in the acute MPTP model (Kurkowska‐Jastrzebska et al., 1999b) and it was necessary to see if this is also true for the sub‐acute model used in this study. Quantitative RT‐PCR showed a significant increase in MHC II β‐chain mRNA in the ventral midbrain one day after MPTP administration compared to saline‐treated mice (*P* = 0.011 ANOVA, Dunnett's post hoc test; Fig. 1A), when normalized to β‐actin levels (β‐actin levels were unchanged by MPTP treatment, *P* = 0.112 Kruskal Wallis test). The increase in MHC II mRNA levels correlated with an increase in MHC II protein one day after MPTP administration which reached statistical significance two days after MPTP treatment (*P* = 0.031 compared to saline, ANOVA, Dunnett's post hoc test; Fig. 1B). The two chains of MHC II, α and β, were dissociated under the conditions used for the Western blots, the bands representing both chains were analyzed together. MHC II protein levels in the striatum were increased at 14 and 21 days after MPTP treatment (*P* = 0.031 at 14 days and *P* = 0.008 at 21 days compared to saline, ANOVA, Dunnett's post hoc test; Fig. 1C), whilst MHC II protein levels in the cerebellum were unchanged by MPTP treatment (*P* = 0.734 ANOVA; Fig. 1D).

Following toxic insults MHC II is reported to be upregulated on both microglia and astrocytes (Kurkowska‐Jastrzebska et al., 1999a; Wong et al., 1984) so the immunohistological localisation of MHC II in the SNpc was determined by fluorescent double‐labelling using TH as a marker for dopaminergic cells, GFAP as a marker for astrocytes and Iba1 as a marker for microglia. MHC II was found to co‐localize with GFAP (Fig. 1E iv‐vi) indicating its presence in astrocytes. MHC II also co‐localized with a subset of Iba1 positive microglia (Fig. 1E vii‐ix).

### Genetic Ablation of MHC II Provides Protection Against MPTP Toxicity

**Figure 2:**
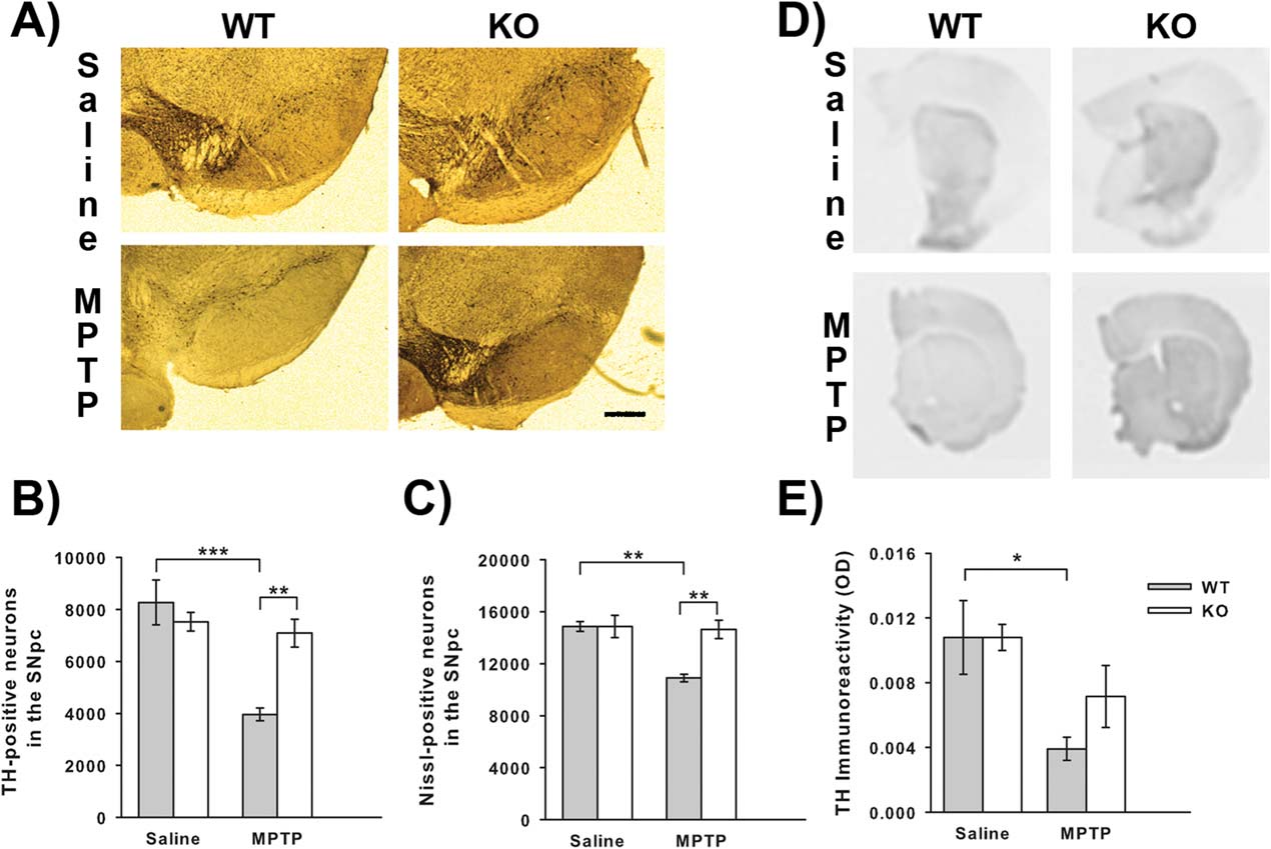
Effect of genetic ablation of MHC II on MPTP neurotoxicity. MHC II null mice show attenuation of MPTP‐induced neuronal loss. Representative micrographs of TH and Nissl stained sections (Scale bar is 200 μm) (**A**). MPTP treatment induced loss of both TH‐positive neuron (**B**) and Nissl‐positive neuron (**C**) numbers in wild‐type mice and this loss was reduced in MHC II null mice. No differences were detected in striatal TH‐immunoreactivity (**D** and **E**) between wild‐type and MHC II null mice. Data are mean ± SEM, *n* = 4–5 mice per group. *P < 0.05; **P < 0.01; ***P < 0.001; ANOVA with student Newman‐Keuls post hoc test for TH‐positive neurons and TH‐immunoreactivity; Kruskal Wallis with Mann Whitney‐U tests for Nissl‐positive neurons (WT—wild‐type; KO—knock‐out (MHC II null); TH‐tyrosine hydroxylase). [Color figure can be viewed in the online issue, which is available at wileyonlinelibrary.com.]

Having determined that MHC II is expressed in the SNpc and altered by MPTP treatment the impact of the absence of MHC II on MPTP toxicity was examined. Treatment with MPTP induces dopaminergic neuron death and this was the case for wild‐type mice that showed a significant reduction in TH‐positive neurons compared to saline‐treated mice (*P* < 0.001 ANOVA, Student Newman Keuls post hoc test; Fig. 2A,B). However, MHC II null mice treated with MPTP did not show any significant reductions in dopaminergic neuron number compared to saline‐treated mice. No significant differences were seen in dopaminergic neuron number between wild‐type and MHC II null mice treated with saline. The same situation was seen with Nissl neuron numbers, with MPTP reducing the number of Nissl positive neurons in wild‐type mice only (*P* = 0.008 Kruskal Wallis, Mann Whitney‐U post hoc test compared to saline‐treated wild‐type mice; Fig. 2C). In the striatum dopaminergic nerve terminals were partially protected in MHC II null mice treated with MPTP. In wild‐type mice MPTP administration reduced striatal TH‐immunoreactivity (*P* = 0.036 ANOVA, Student Newman Keuls post hoc test; Fig. 2E). However, in MHC II null mice MPTP‐induced reduction in TH‐immunoreactivity was less pronounced (not significantly compared to wild‐type mice) and did not significantly differ from saline‐treated mice. The neuroprotective effect of MHC II ablation did not extend into functional protection as there were no differences in the levels of dopamine and its metabolites (Table 1) between wild‐type and MHC II null mice treated with MPTP. Levels of dopamine and DOPAC were both reduced by MPTP treatment in wild‐type (dopamine—*P* < 0.001 Kruskal Wallis, Mann Whitney‐U post hoc test; DOPAC—*P* < 0.001 Kruskal Wallis, Mann Whitney‐U post hoc test) and MHC II null mice (dopamine—*P* < 0.001 Kruskal Wallis, Mann Whitney‐U post hoc test; DOPAC—*P* = 0.010 Kruskal Wallis, Mann Whitney‐U post hoc test) compared to saline‐treated mice.

**Table 1:** Effect of Genetic Ablation of MHC II on Striatal Dopamine and DOPAC Levels

	Saline		MPTP	
	WT	KO	WT	KO
Dopamine (ng/mg wet tissue)	7.32 ± 1.42	9.17 ± 1.21	1.27 ± 0.30***	1.74 ± 0.34***
DOPAC (ng/mg wet tissue)	0.76 ± 0.08	1.52 ± 0.35	0.24 ± 0.05***	0.39 ± 0.11*

No difference is seen between wild‐type and MHC II null mice in their sensitivity to MPTP toxicity as measured by reduction in dopamine and DOPAC levels. Data are mean ± SEM, *n* = 5–8 mice per group.

*P < 0.05.

***P < 0.001 compared to appropriate saline‐treated group (Kruskal‐Wallis test with Mann Whitney U‐post hoc tests; WT—wild‐type, KO—knock‐out (MHC II null mice)).

### Genetic Ablation of MHC II Reduces MPTP‐Induced Microgliosis and Astrogliosis

**Figure 3:**
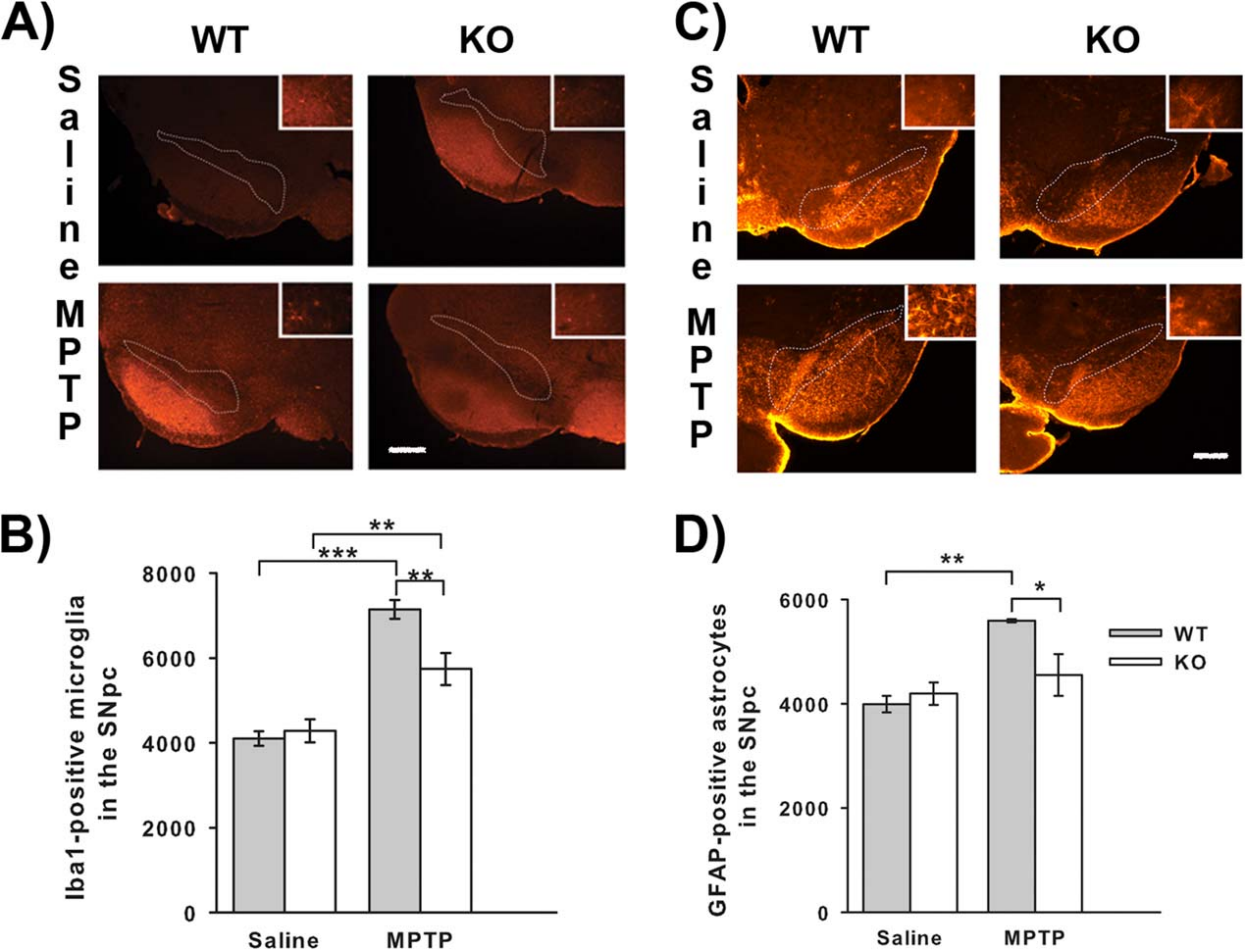
Effect of genetic ablation of MHC II on MPTP‐induced reactive microgliosis and astrogliosis. MHC II null mice show attenuation of MPTP‐induced microgliosis and astrogliosis. Representative micrographs of Iba1 stained sections one day after MPTP (Scale bar is 200μm) (**A**). MPTP‐induced reactive microgliosis was present in both wild‐type and MHC II null mice, but was reduced in MHC II null mice compared to wild‐type mice (**B**). Representative micrographs of GFAP stained sections two days after MPTP (Scale bar is 200μm) (**C**). MPTP‐induced reactive astrogliosis was attenuated in MHC II null mice compared to wild‐type mice (**D**). Data are mean ± SEM, *n* = 4–5 mice per group. *P < 0.05; **P < 0.01; ***P < 0.001; ANOVA with student Newman‐Keuls post hoc test (WT—wild‐type; KO—knock‐out (MHC II null); Iba1 Ionized calcium‐binding adaptor molecule 1; GFAP‐glial fibrillary acidic protein). [Color figure can be viewed in the online issue, which is available at wileyonlinelibrary.com.]

Administration of MPTP results in reactive gliosis beginning one day after MPTP treatment for microglia and two days after MPTP for astrocytes (Kohutnicka et al., 1998). To see if the degree of reactive gliosis was altered in MHC II null mice, the number of Iba1‐positive microglia and the number of GFAP‐positive astrocytes were stereologically counted one and two days after MPTP treatment respectively. MPTP treatment significantly increased the number of Iba1‐positive microglia in both wild‐type (*P* < 0.001 ANOVA, Student Newman Keuls post hoc test; Fig. 3A,B) and MHC II null mice (*P* = 0.001 ANOVA, Student Newman Keuls post hoc test) compared to saline‐treated controls. However, MPTP treated MHC II null mice demonstrated less Iba1‐positive microglia than MPTP treated wild‐type mice (*P* = 0.002 ANOVA, Student Newman Keuls post hoc test). The number of Iba1‐positive microglia did not differ between wild‐type and MHC II null mice treated with saline. The number of GFAP‐positive astrocytes was also increased by MPTP in wild‐type mice (*P* = 0.007 ANOVA, Student Newman Keuls post hoc test; Fig. 3C,D), however, this increase was significantly attenuated in MHC II null mice (*P* = 0.016 ANOVA, Student Newman Keuls post hoc test). The number of GFAP‐positive astrocytes in MPTP treated MHC II null mice did not differ from that of saline‐treated mice and the number of GFAP‐positive astrocytes did not differ between wild‐type and MHC II null mice treated with saline.

### Genetic Ablation of MHC II Changes Cytokine Responses to MPTP

**Figure 4:**
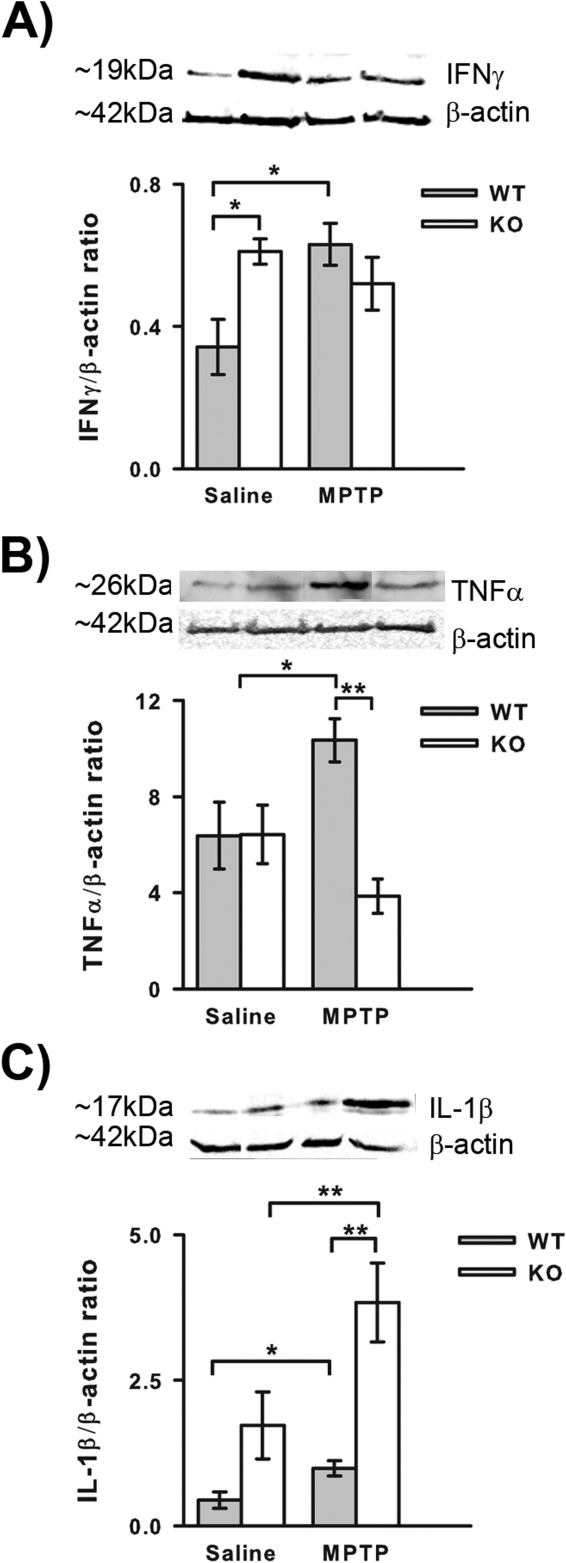
Effect of genetic ablation of MHC II on cytokine responses one day after MPTP treatment. IFN‐γ protein levels are increased in MHC II null mice treated with saline compared to wild‐type mice, but MPTP treatment did not induce an increase in IFNγ in MHC II null mice as it did in wild‐type mice (**A**). MPTP‐induced increases in TNFα protein levels are attenuated in MHC II null mice (**B**). Interleukin‐1β protein levels are increased in MHC II null mice treated with saline compared to wild‐type mice, MPTP treatment increased IL‐1β protein levels in both MHC II null and wild‐type mice. Data are mean ± SEM, *n* = 4–5 mice per group. *P < 0.05; ** *P* < 0.01; ANOVA with student Newman‐Keuls post hoc test (WT—wild‐type; KO—knock‐out (MHC II null); IFNγ—interferon‐γ; TNFα—tumour necrosis factor‐α; IL‐1β—interleukin‐1β).

As MHC II ablation reduced both MPTP‐induced dopaminergic loss and reactive gliosis processes involving cytokine production, alterations in cytokine responses to MPTP administration were assessed. The cytokines chosen for assessment were interferon‐γ (IFNγ), tumour necrosis factor‐α (TNFα) and interleukin‐1β (IL‐1β) as these cytokines are linked to both MHC II induction (Dong and Benveniste, 2001) and PD pathogenesis (Mogi et al., 1994a, 1994b; Mount et al., 2007). Saline‐treated MHC II null mice had higher levels of IFNγ than saline‐treated wild‐type mice (*P* = 0.040 ANOVA, Student Newman Keuls post hoc test; Fig. 4A). In wild‐type mice MPTP induced an increase in IFNγ levels (*P* = 0.035 ANOVA, Student Newman Keuls post hoc test) but this MPTP‐induced increase was not seen in the MHC II null mice. TNF‐α levels were also increased by MPTP treatment in wild‐type mice (*P* = 0.018 ANOVA, Student Newman Keuls post hoc test; Fig. 4B) and this increase was attenuated in MHC II null mice (*P* = 0.001 ANOVA, Student Newman Keuls post hoc test), where the levels of TNFα did not differ from saline‐treated mice. The levels of TNFα levels did not differ between wild‐type and MHC II null mice treated with saline. IL‐1β was increased by MPTP treatment in both wild‐type and MHC II null mice compared to saline‐treated mice (wild‐type—*P* = 0.036 ANOVA, Student Newman Keuls post hoc test; MHC II null—*P* = 0.009 ANOVA, Student Newman Keuls post hoc test; Fig. 4C). The magnitude of increase was greater in MHC II null mice compared to wild‐type mice (*P* = 0.002 ANOVA, Student Newman Keuls post hoc test).

## Discussion

In this study, MHC II levels in the ventral midbrain, the area containing the SNpc, were increased soon after MPTP treatment. This is in contrast to previous work (Kurkowska‐Jastrzebska et al., 1999b) where MHC II levels were not increased until three days after MPTP with peak expression 14 days after MPTP. However, (Kurkowska‐Jastrzebska et al., 1999b) focussed on MHC II‐positive microglia whilst the results from the current study included a heterogeneous mixture of cell types. This study demonstrated that MHC II also co‐localized with the astrocytic marker GFAP, as previously shown (Kurkowska‐Jastrzebska et al., 1999a; Wong et al., 1984), suggesting that the increase in MHC II levels may arise from astrocytes. The increase in MHC II protein levels seen coincides with increased GFAP expression after MPTP treatment (Kohutnicka et al., 1998) adding further support to astrocytes potentially being the predominant source of the MHC II upregulation. In the striatum MHC II levels were not elevated until 14 days after MPTP treatment which is consistent with previous work (Kurkowska‐Jastrzebska et al., 1999b). This increase occurs after the peak of dopaminergic neuron death (Jackson‐Lewis et al., 1995) and is more likely to be a result from the degeneration of the dopaminergic terminals rather than being an active component of the degeneration.

Genetic ablation of MHC II provided neuroprotection to the cell bodies of dopaminergic neurons in the SNpc against MPTP toxicity, but this did not extend to functional protection of the dopaminergic nerve terminals in the striatum. This is consistent with early upregulation of MHC II in the ventral midbrain, suggesting that this upregulation of MHC II contributes to dopaminergic neuron death. A similar pattern of neuroprotection was seen in mice null for the T‐cell receptor or CD4 (Brochard et al., 2009) and as MHC II is important for activation of T‐cells (Dong and Flavell, 2001) these data suggest that an adaptive immune response is involved in MPTP‐induced dopaminergic neuron death. Interestingly, CD4^+^ T‐helper (T_H_) cells, particularly T_H_1 cells, are increased in the peripheral blood of PD patients (Baba et al., 2005) and T_H_1 cells recruit CD8^+^ cytotoxic T cells which are the predominant T‐cell infiltrate in the brain in PD patients at post‐mortem and in MPTP‐lesioned animals (Brochard et al., 2009), providing further support for an adaptive CD4^+^ T_H_ cell mediated immune response in PD. Further evidence is emerging for a possible role of the adaptive immune in the development of Parkinson's disease as toll‐like receptor 4 (TLR4) gene polymorphisms have been linked to sporadic Parkinson's disease (Zhao et al., 2015) and α‐synuclein can induce the up‐regulation of TLRs (Beraud et al., 2011). Additionally, ablation of TLR4 provided neuroprotection in the MPTP‐model of PD (Noelker et al., 2013).

A direct role for MHC II in the immune/inflammatory response to MPTP is further supported by the significant reduction of microgliosis in MHC II null mice compared to wild‐type mice. As microgliosis was not completely attenuated in MHC II null mice this suggests that some of the microgliosis seen following MPTP treatment is MHC II independent. Initial microglial activation is likely to be MHC II independent as this occurred before MHC II upregulation (Araneda et al., 1980) and following activation MHC II is upregulated (Kreutzberg, 1996). The reduction in microgliosis in MHC II null mice may be the result of a lack of infiltration/activation of CD4^+^ T‐cells and the release of IFNγ, indeed IFNγ levels were not upregulated by MPTP treatment in MHC II null mice as they were in wild‐type mice. IFN‐γ is known to be an important activator of microglia and to date no CNS source for IFNγ has been identified (Lynch, 2009) suggesting that infiltrating cells (i.e. T cells) are the most likely source of IFNγ. Thus the reduction in microgliosis could result from the attenuation of T‐cell infiltration and IFNγ production. However, microgliosis was assessed one day after MPTP treatment and significant CD4^+^ T‐cell infiltrates are not seen until two days after MPTP administration (Björklund et al., 1986), but Brochard and coworkers used the acute MPTP regime compared to the sub‐acute regime used here. Differences between these regimes have been reported (Luchtman et al., 2009), which means CD4^+^ T‐cell infiltration may occur earlier in the sub‐acute regime. The total MPTP dose of the acute regime, 80 mg/kg, was reached by the third day of dosing in the sub‐acute regime suggesting that CD4^+^ T‐cell infiltration may occur by the time used to assess microgliosis, but it is not possible to conclusively attribute the reduction in microgliosis to a lack of CD4^+^ T‐cell infiltrates without further work.

Also proteins like alpha‐synuclein (α‐synuclein), which has long been implicated in the pathogenesis of PD (Polymeropoulos et al., 1997) could act as both modulators of glial functions and as antigens themselves activating the peripheral and central immune system (Harms et al., 2013; Reynolds et al., 2008; Sanchez‐Guajardo et al., 2015). Thus in PD itself, α‐synuclein itself might be an antigen used by MHC II during antigen presentation and thus leading to the observed glial infiltration in PD (Hunot and Hirsch, 2003). However it has been also demonstrated that MHC II is upregulated in Parkinson brains, and was not linked to the presence of Lewy‐bodies, indicating, that α‐synuclein might only play a minor role in the recruitment of MHC II positive microglia, and invasion occurs due to the neuronal injury and the associated phagocytosis (Imamura et al., 2003; McGeer et al., 1988).

Astrogliosis was completely attenuated in MHC II null mice suggesting that astrocytic activation following MPTP treatment involves an MHC II dependent process. This may be a lack of infiltrating T‐cells and IFNγ release, as IFNγ in combination with TNFα is an important activator of astrocytes (Dong and Benveniste, 2001). Activated microglia can increase astrocyte number *in vitro* (Rohl et al., 2007) and the astrogliosis seen in PD/MPTP has an astrogenic component (Kohutnicka et al., 1998). Taken together this suggests that the attenuation of astrogliosis in MHC II null mice was just a downstream effect of reduced microgliosis. However, there is evidence that astrogliosis can occur independently of microgliosis following MPTP treatment, as interleukin‐6 null mice are more vulnerable to MPTP toxicity (Bolin et al., 2002) and microgliosis was completely attenuated in these mice whilst astrogliosis was unaffected (Cardenas and Bolin, 2003). A role for astrogliosis independent of microgliosis receives a degree of support from the current study as astrocytes were the major source of MHC II expression. This suggests that it is actually astrocytes that interact with infiltrating CD4^+^ T‐cells leading to cytokine production and reactive gliosis, further supported by the integral role of astrocytes in the blood‐brain barrier (Prat et al., 2001). However, there is conflicting evidence whether astrocytes express the co‐stimulatory molecules, B7 and CD40, required to activate infiltrating CD4^+^ T‐cells (Aloisi et al., 1998; Nikcevich et al., 1997; Tan et al., 1998). Further work is needed to determine the importance of MHC II‐positive astrocytes in MPTP toxicity. Unfortunately it is difficult to assess the role of astrocytes in dopaminergic neuron death as astrocytes are required for the biotransformation of MPTP to its toxic metabolite MPP^+^ (Ransom et al., 1987), and interfering with astrocytes function has been shown to reduce dopaminergic neuron loss via reductions in MPP^+^ production (Takada et al., 1990). As both astrogliosis and microgliosis were reduced in MHC II null mice it is not possible to determine which plays a more important role in the pathogenesis of dopaminergic neuron loss. It is likely that both contribute to dopaminergic neuron loss as both astrocytes and microglia produce pro‐inflammatory cytokines (Dong and Benveniste, 2001; Hanisch, 2002). However, some of these pro‐inflammatory cytokines, especially IFNγ, may be derived from infiltrating CD4^+^ T‐cells. Irrespective of their source, all these cytokines are increased in PD patients (Mogi et al., 1994a, 1994b; Mount et al., 2007) and are documented to have negative impacts on MPTP toxicity (Mount et al., 2007; Ferger et al., 2004). Indeed IFNγ null mice showed significant attenuation of MPTP‐induced loss of dopaminergic neurons together with ablation of microgliosis (Mount et al., 2007), suggesting that IFNγ activation of microglia is important in MPTP toxicity. TNF‐α null mice also showed attenuation of MPTP toxicity, but this effect was confined to the striatum (Ferger et al., 2004). As the protection from MPTP toxicity derived from the ablation of MHC II did not extend to the striatum it would suggest that IFNγ is more important for dopaminergic neuron death in the SNpc. In contrast to IFNγ and TNFα MPTP‐induced increases in IL‐1β levels were seen in both wild‐type and MHC II null mice, which suggests that regulation of this cytokine is independent of the MHC II pathway. Indeed IL‐1 inhibition reduced dopaminergic neurodegeneration induced by 6‐hydroxydopamine or lipopolysaccharide treatment without downregulating microglial activation (Pott Godoy et al., 2008). The lack of impact of MHC II ablation on IL‐1β levels may also be due to the significant degree of microgliosis that still occurred as microglia are an important source of IL‐1β following insults (Hanisch, 2002). Furthermore chronic, systemic administration of IL‐1 together with 6‐OHDA increases dopaminergic neuron loss and the number of MHC II‐positive cells (Pott Godoy et al., 2008). These data suggest that IL‐1β has a role in regulating MHC II responses after dopaminergic toxic insults, and mice null for the IL‐1 receptor 1 cannot activate CD4^+^ T‐cells (Eriksson et al., 2003). The IL‐1 β is probably derived from infiltrating MHC II‐monocytes.

In conclusion this study has shown that MHC II upregulation is important for dopaminergic neuron death by a mechanism that involves reactive gliosis. This study also further supports the presence of an adaptive immune response in PD pathogenesis, and it suggests that astrocytes, as well as microglia, play an important part in this response. Further work is required to delineate the role of cytokines in this adaptive immune response, as the current study shows that IL‐1β may play an important role in regulating MHC II responses, whilst IFNγ and TNFα appear to be important for inflammatory processes downstream of MHC II activation in reactive gliosis. It will be interesting to further explore the molecular mechanisms underlying the adaptive immune response seen in this study and their relevance to PD pathogenesis.
